# A novel far-red fluorescent xenograft model of ovarian carcinoma for preclinical evaluation of HER2-targeted immunotoxins

**DOI:** 10.18632/oncotarget.5130

**Published:** 2015-09-05

**Authors:** Tatiana Zdobnova, Evgeniya Sokolova, Oleg Stremovskiy, Dmitry Karpenko, William Telford, Ilya Turchin, Irina Balalaeva, Sergey Deyev

**Affiliations:** ^1^ Lobachevsky State University of Nizhny Novgorod, Nizhny Novgorod 603950, Russia; ^2^ Institute of Bioorganic Chemistry of The Russian Academy of Sciences, Moscow 117997, Russia; ^3^ National Cancer Institute, National Institutes of Health, Bethesda, MD 20892, USA; ^4^ Institute of Applied Physics of The Russian Academy of Sciences, Nizhny Novgorod, 603950 Russia

**Keywords:** xenograft tumor model, far-red fluorescent protein, ovarian carcinoma, HER2, immunotoxin

## Abstract

We have created a novel fluorescent model of a human ovarian carcinoma xenograft overexpressing receptor HER2, a promising molecular target of solid tumors. The model is based on a newly generated SKOV-kat cell line stably expressing far-red fluorescent protein Katushka. Katushka is most suitable for the *in vivo* imaging due to an optimal combination of high brightness and emission in the “window of tissue transparency”. The relevance of the fluorescent model for the *in vivo* monitoring of tumor growth and response to treatment was demonstrated using a newly created HER2-targeted recombinant immunotoxin based on the 4D5scFv antibody and a fragment of the *Pseudomonas* exotoxin A.

## INTRODUCTION

Xenograft models of human cancer are indispensable tools for evaluation of efficacy of novel anti-tumor therapeutic agents. With the development of the fluorescent protein technology, experimental fluorescent tumor models have become widespread [1], allowing easy and inexpensive detection and quantification of a tumor using optical fluorescence imaging. As compared with MRI, PET, and SPECT, this method is rapid, has a high throughput, does not require contrasting agents or expensive imaging equipment [2, 3], and provides a non-invasive, highly specific, and high-resolution monitoring of tumor growth and metastasis in an individual animal *in vivo* over a prolonged period of time. Moreover, fluorescent tumor models make it possible to take into account only tumor cells without stromal cells, connective-tissue capsule and vessels of host nature [4]. This renders the assessment of therapeutic effects of anti-tumor agents more accurate and reproducible than commonly applied vernier caliper measurements, especially in the case of small or deeply laying tumors [5].

Owing to the considerable progress in molecular oncology, a variety of tumor molecular targets has become known to date. This led to implementation of a new class of targeted anticancer agents from therapeutic monoclonal antibodies to sophisticated stimuli-controlled particles-based theranostics agents [6]. Evaluation of the therapeutic effects of the newly engineered targeted agents requires the development of adequate tumor models expressing appropriate targets.

The human epidermal growth factor receptor-2 (HER2) is a well-known diagnostic marker and advanced molecular target for a targeted therapy of cancer [7]. This receptor is overexpressed in a range of tumor types including but not limited to breast, ovarian, endometrial, colon, prostate, cervical, and non-small-cell lung cancer. HER2 is implicated in disease initiation and progression, is associated with poor prognosis and may predict the response to chemotherapy and hormonal therapy [8]. The development of suitable models that allow visualization and quantification of antineoplastic efficacy of HER2-targeted agents *in vivo* is necessary to carry out preclinical evaluations. Recently, a HER2-expressing mouse breast cancer cell line 4T1 transfected with GFP has been used for intraoperative imaging of metastatic lymph nodes [9].

In this study we created a novel fluorescent xenograft model characterized by the HER2 overexpression and emission in the far-red region of the spectrum. We demonstrated the relevance of the fluorescent model for the *in vivo* evaluation of anti-tumor efficacy of novel HER2-targeted recombinant immunotoxin and commonly used chemotherapeutic agent cisplatin.

## RESULTS

### Generation and characterization of fluorescent cell line overexpressing HER2 and subsequent xenograft tumor model

**Figure 1:**
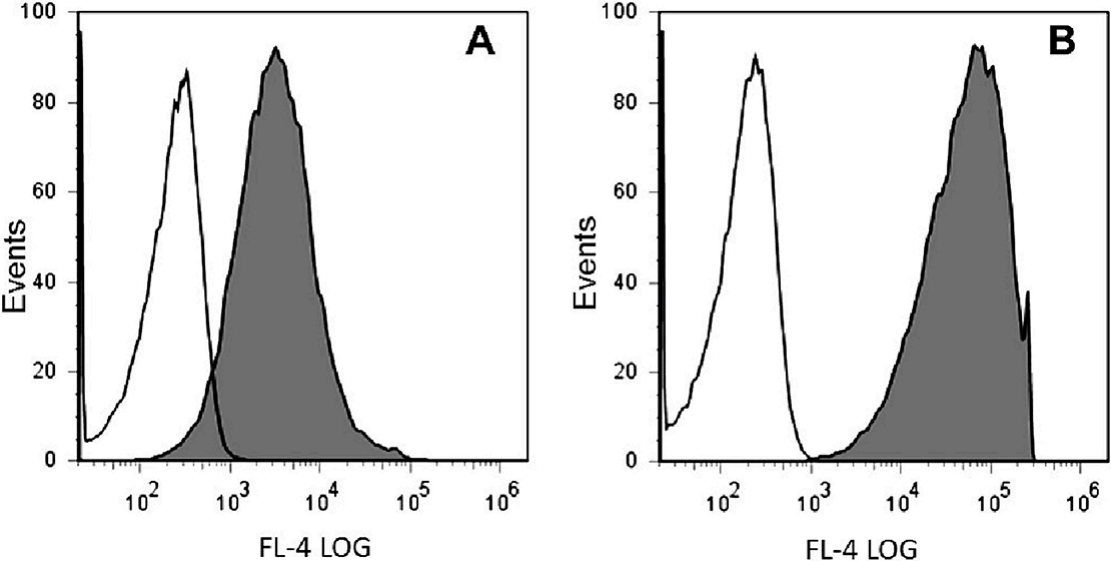
Sorting of the SKOV-kat cells based on Katushka fluorescence **A.** Initial Katushka-positive SKOV-kat population (grey histogram) versus parental SKOV-3 cells (white histogram). **B.** Sorted SKOV-kat after three sort/expansion cycles (grey histogram) versus parental SKOV-3 cells (white histogram).

**Figure 2:**
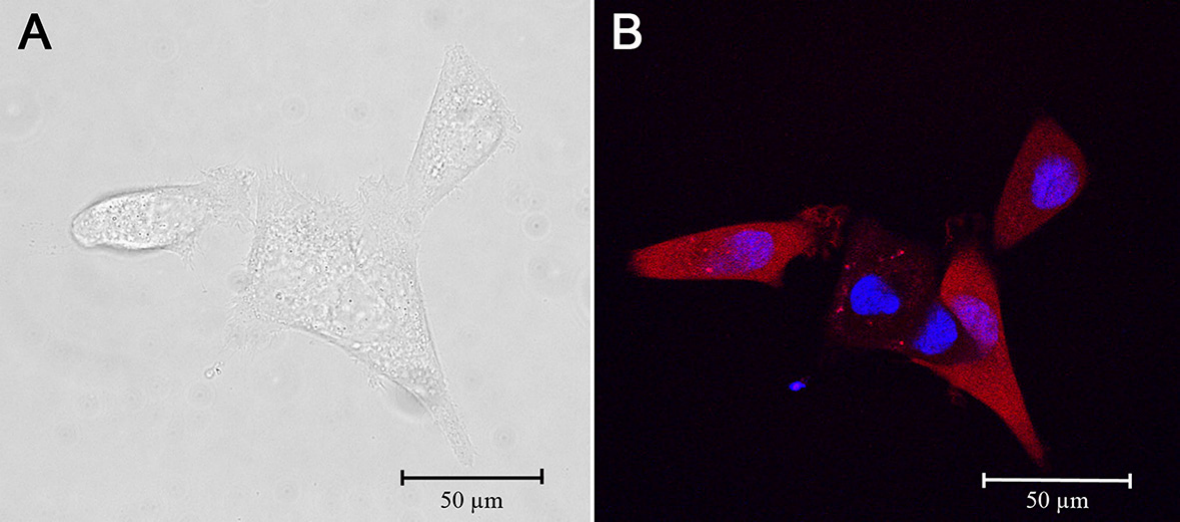
Visualization of SKOV-kat cells **A.** SKOV-kat cells in transmitted light; **B.** Fluorescence of SKOV-kat cells expressing protein Katushka (red) visualized by confocal microscopy; nuclei are stained with Hoechst 33258 (blue).

Human ovarian adenocarcinoma cell line SKOV-3 was used as parental for generation of a novel fluorescent cell line overexpressing HER2. The SKOV-3 cell line was stably transfected with the fluorescent protein Katushka gene. To improve their fluorescence properties, the transfected cells were sorted three times following multiple expansions and the cells with the highest expression of the fluorescent protein were collected for further expansion. By repeating the sorts, the mean fluorescence level was increased up to 20–30 times of the fluorescence of the original transfectants (Fig. 1). The obtained cell line was named SKOV-kat (Fig. 2).

**Figure 3:**
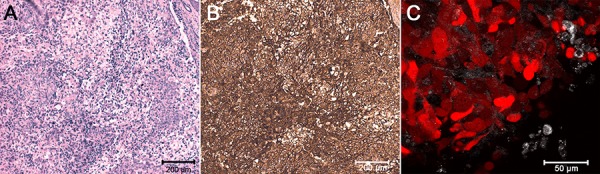
Characterization of tumor xenograft **A.** Staining of a tumor tissue section with hematoxilin and eosin (H&E). **B.** Immunohistochemical staining of a tumor tissue section with HercepTest (Dako); strong brown coloring corresponds to HER2 overexpression. **C.** Confocal image of tumor tissue; red color, TurboFP635 fluorescence. The image acquisition parameters were the same as in experiments on cells (described in the Materials and Methods section).

SKOV-kat cells implanted subcutaneously in the subscapular area of BALB/c nude mice formed a fluorescing tumor with high cellularity and thin interlayers of connective tissue; ICH revealed HER2 overexpression (+++) in the tumor tissue (Fig. 3). Fluorescence signal from cancer cells was easily detected *in vivo* by epifluorescence imaging immediately after the injection and then during the tumor development. Strong correlation of tumor volume calculated on vernier caliper measurements and integral fluorescence of tumor (Fig. S1) made it possible to quantify tumor progression and the response to treatment by whole-body imaging.

### Immunotoxin construction, purification and characterization

**Figure 4:**
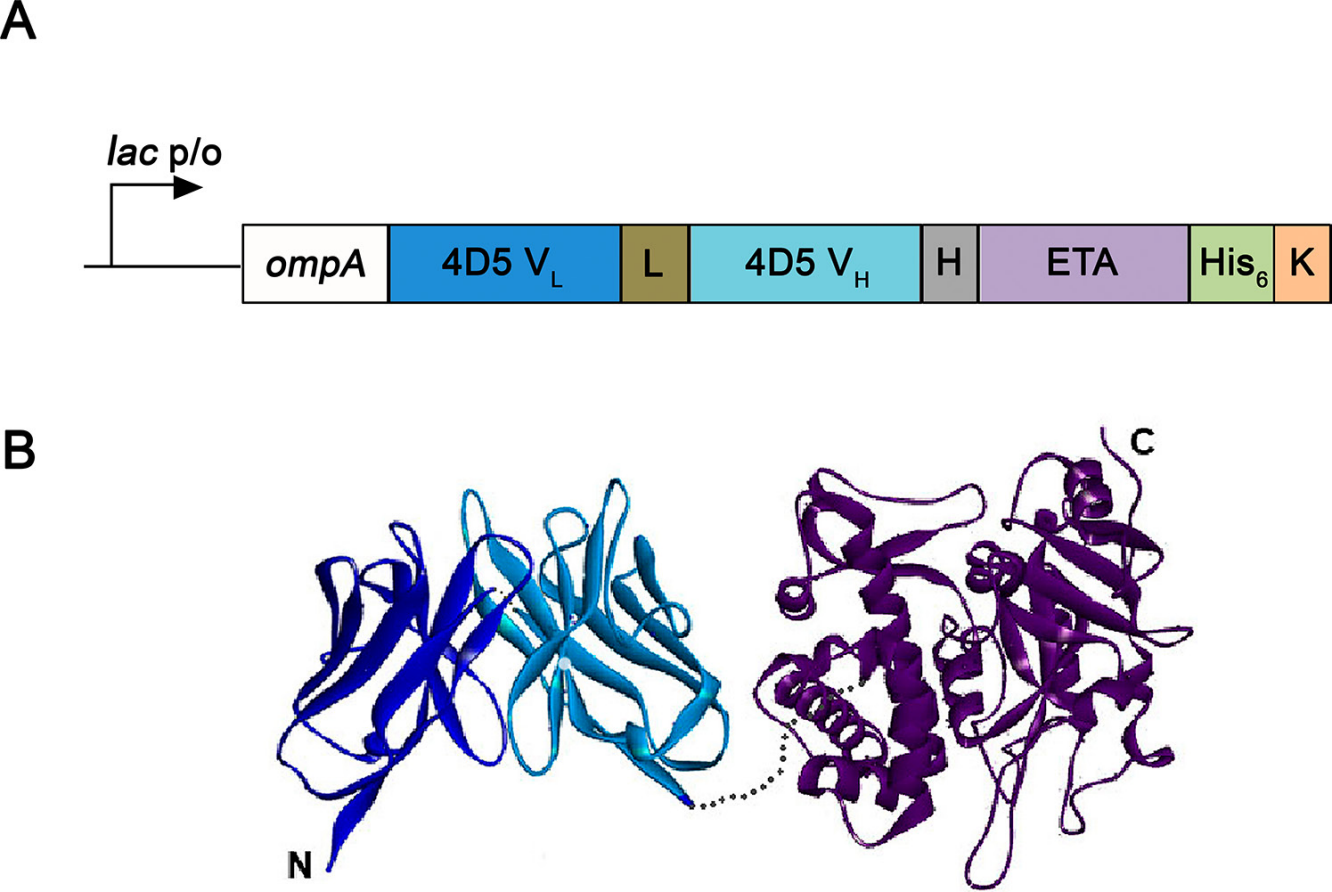
Design of recombinant immunotoxin 4D5scFv-ETA **A.** Gene construct encoding immunotoxin 4D5scFv-ETA. *OmpA*, a signal peptide for directed secretion of the recombinant protein to the E. coli periplasm. 4D5 V_L_ (blue), light chain of monoclonal antibody 4D5scFv; L (brown), designed linker (21 a.a.); 4D5 V_H_ (turquoise), heavy chain of 4D5scFv monoclonal antibody; H (gray), hinge-like linker (16 a.a.); ETA (purple), 252–613 a.a. of the Pseudomonas exotoxin A; His_6_ (green), C-terminal cluster of 6 His residues (His6-tag); K (orange), KDEL sequence. The fusion gene is under control of the lac promoter. **B.** Molecular model (ribbon representation) of 4D5scFv-ETA created using DS Viewer Pro 5.0 software, based on the X-ray crystal structure of the wild-type ETA (Protein Data Bank code: 1IKQ) and 4D5scFv (Protein Data Bank code: 1FVC). Colors are as in Fig. 4A.

The scFv fragment of the monoclonal antibody 4D5 containing light (V_L_) and heavy (V_H_) chain variable domains was fused to a truncated *Pseudomonas* exotoxin A (a.a. 252–613, herein referred to as ETA) representing translocation domain II, domain Ib and domain III which catalyzes the ADP ribosylation and inactivation of eucaryotic elongation factor 2 (EEF2), thus arresting the protein synthesis and leading to cell death. It has been previously shown that natural N-terminus of 4D5scFv is important for proper antibody function [10]. Therefore, the nucleotide sequence of hybrid gene encoding ETA fragment was placed at the C-terminus of the 4D5scFv encoding sequence (Fig. 4A). The targeting (4D5scFv) and cytotoxic (ETA) moieties were connected via a 16-amino-acid flexible linker derived from the mouse IgG3 hinge region [11] that prevents spatial interference between the two domains. To increase the cytotoxic potency of the toxin, KDEL sequence at the C-terminus of the ETA fragment was added [12]. A synthetic cluster of six His residues at the C-terminus facilitated purification of the protein via Ni^2+^ affinity chromatography. A 3D model of the mature 4D5scFv-ETA immunotoxin molecule is shown in Fig. 4B.

The resulting fusion protein 4D5scFv-ETA was produced in E. coli and purified as described in Materials and Methods. The obtained protein was of the expected molecular weight and homogeneity according to SDS-PAGE (Fig. S2).

To make sure that binding affinity of 4D5scFv was conserved in the fused protein, dissociation constants (Kd) of the 4D5scFv-ETA immunotoxin were determined on immobilized recombinant protein p185HER2-ECD by surface plasmon resonance with the BIAcore analyzer. The fused 4D5scFv showed binding characteristics comparable in specificity and affinity to those of the parental antibody; Kd of 4D5scFv-ETA (∼6.8 nM) was similar to that of 4D5scFv (Kd ∼5.2 nM) (Fig. S3). These results are in agreement with previously published data on affinity values for 4D5scFv determined on cells by RIA (Kd ∼3nM) [13]. Thus, 4D5scFv located on N-terminus of the designed immunotoxin 4D5scFv-ETA retains its specificity and high affinity typical for the parental antibody.

The cytotoxic effect of 4D5scFv-ETA was tested on a number of cell lines with different levels of the HER2 expression: cell lines SKOV-3 and SKOV-kat overexpressing HER2 [14]; HeLa cells with relatively low level of the HER2 expression [15], and HER2-negative CHO cells [16]. Besides, the effects of free 4D5scFv and ETA were evaluated separately to verify the efficacy of 4D5scFv-ETA as a fuse of targeting and cytotoxic modules.

**Figure 5:**
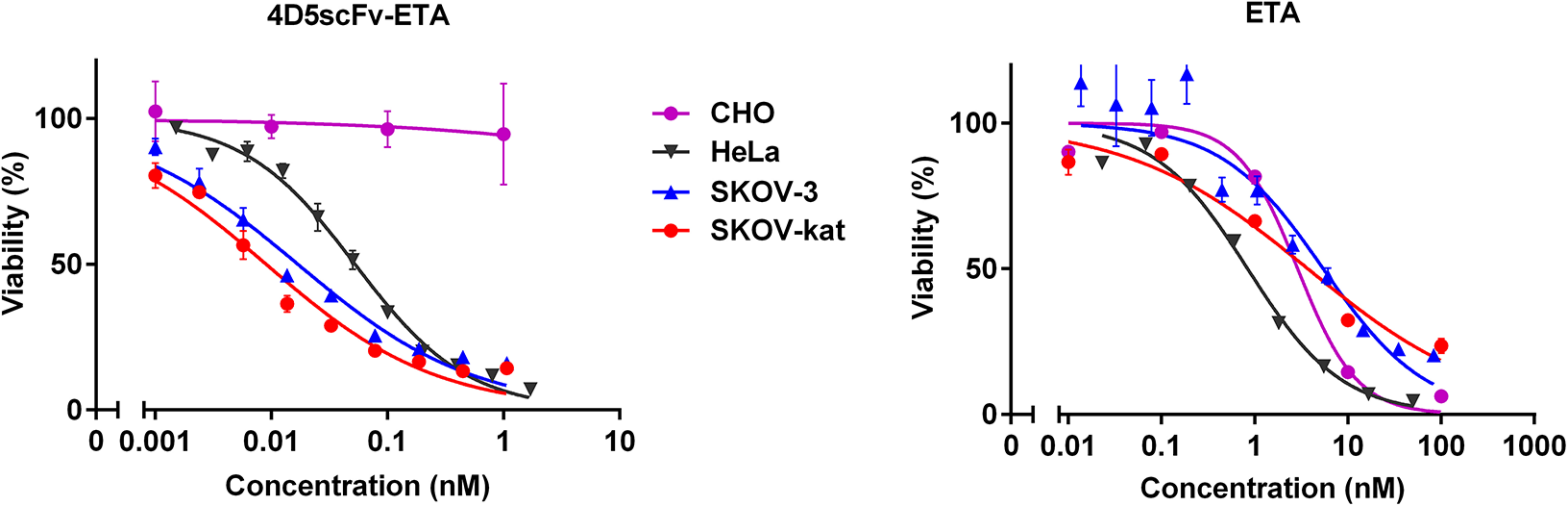
*In vitro* analysis of the 4D5scFv-ETA cytotoxicity Relative viability of HER2-positive cells SKOV-3 (blue line and triangles), SKOV-kat (red line and circles), and HeLa (black line and inverted triangles) and of HER2-negative cells CHO (magenta line and circles) after the treatment with 4D5scFv-ETA or free ETA at different concentrations. Error bars represent the standard error of the mean (SEM) of triplicate wells. The experiments were repeated at least 2 times.

4D5scFv-ETA was highly cytotoxic against the HER2-positive cell lines, and the effect correlated with the expression level of HER2 (Fig. 5). Cytotoxic effect of the immunotoxin on the HER2-negative CHO cells was much less pronounced. To estimate the specificity of the immunotoxin due to its targeting module we calculated the targeting index for each cell line tested (IC_50_ ETA/IC50 4D5scFv-ETA). For HeLa cells with low level of the HER2 expression IC_50_ of free ETA was about 15 times higher than that of 4D5scFv-ETA. For SKOV-3 cells and their derivative SKOV-kat cells used in continued *in vivo* study, the targeting index reached the values of about 388 and 612, respectively (Table 1).

**Table 1:** IC_50_ of immunotoxin 4D5scFv-ETA and free ETA for cell lines with different levels of the HER2 expression

Cell line	IC_50_^a^, nM		Targeting index^b^
	4D5scFv-ETA	ETA	
SKOV-3	**0.017** (0.011 – 0.025)	**6.6** (3.1 – 14.0)	388^c^
SKOV-kat	**0.008** (0.006 – 0.013)	**4.9** (1.3 – 18.4)	612^c^
HeLa	**0.053** (0.045 – 0.061)	**0.8** (0.3 – 2.0)	15^c^
CHO	**8.7** (5.6 – 13.6)	**2.9** (1.8 – 4.6)	0.33

a IC_50_ values are given with 95% confidence interval

b IC_50_ ETA/IC_50_ 4D5scFv-ETA

c statistically different from HER2-negative CHO cells at *P* < 0.0001 (Dunnett's multiple comparisons test)

No cytotoxic effect of 4D5scFv was revealed in any cell culture tested (Fig. S4).

### *In vivo* evaluation of the anti-tumor efficacy of the immunotoxin in the SKOV-kat xenograft model

**Figure 6:**
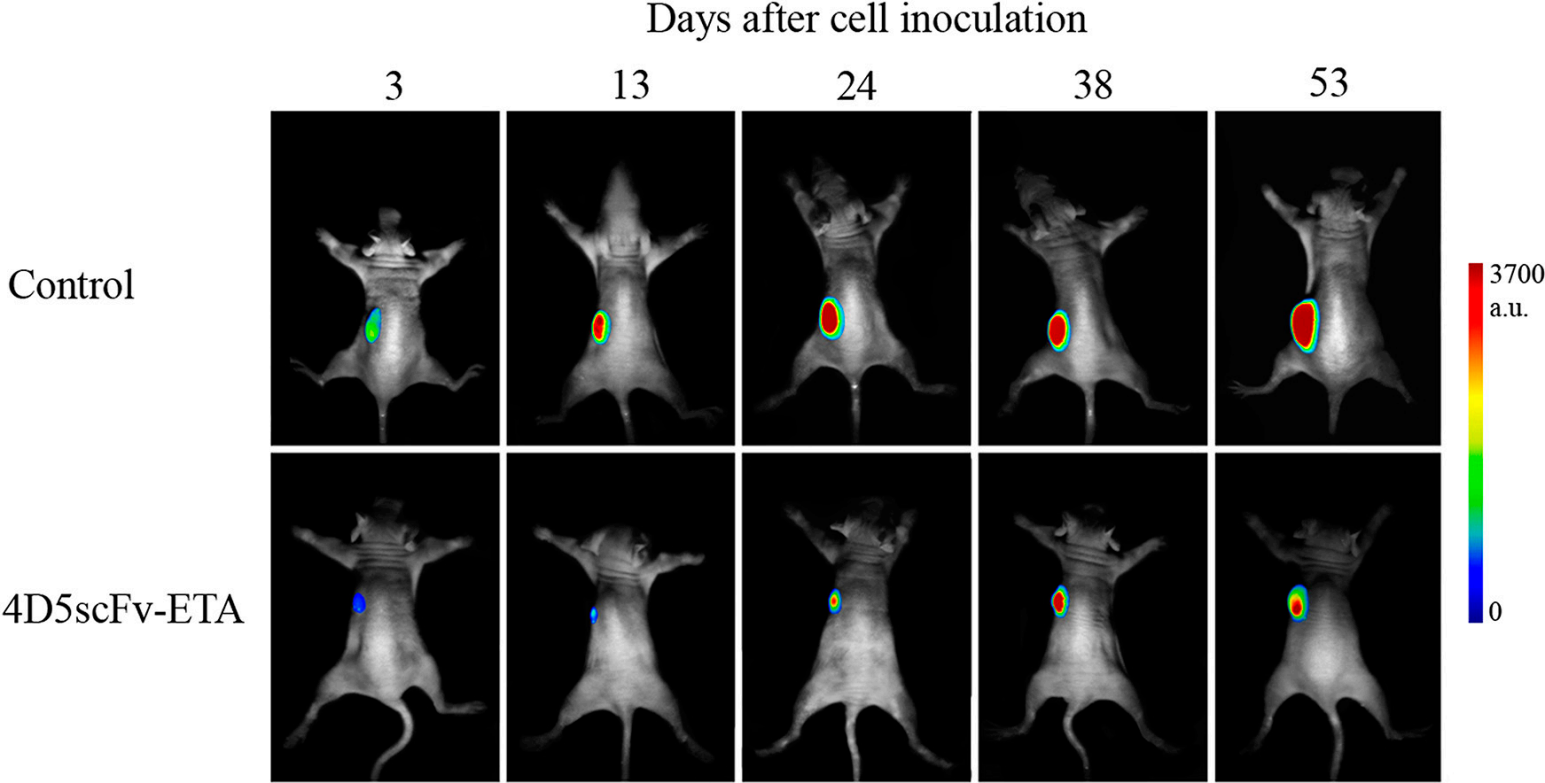
Sequential *in vivo* images of control (injection of PBS) and 4D5scFv-ETA-treated animals The day of inoculation of SKOV-kat cells to animals was set as day 0. At the first day after inoculation mice were treated with 4D5scFv-ETA i.p. at a dose of 50 pmol per animal. *In vivo* 2D images were acquired using whole-body fluorescence imaging setup with planar epi-illumination geometry.

The SKOV-kat xenograft tumor model was used to evaluate the inhibition of tumor growth after the treatment of mice with HER2-targeted immunotoxin (Fig. 6) or chemotherapy drug cisplatin or their combination.

**Figure 7:**
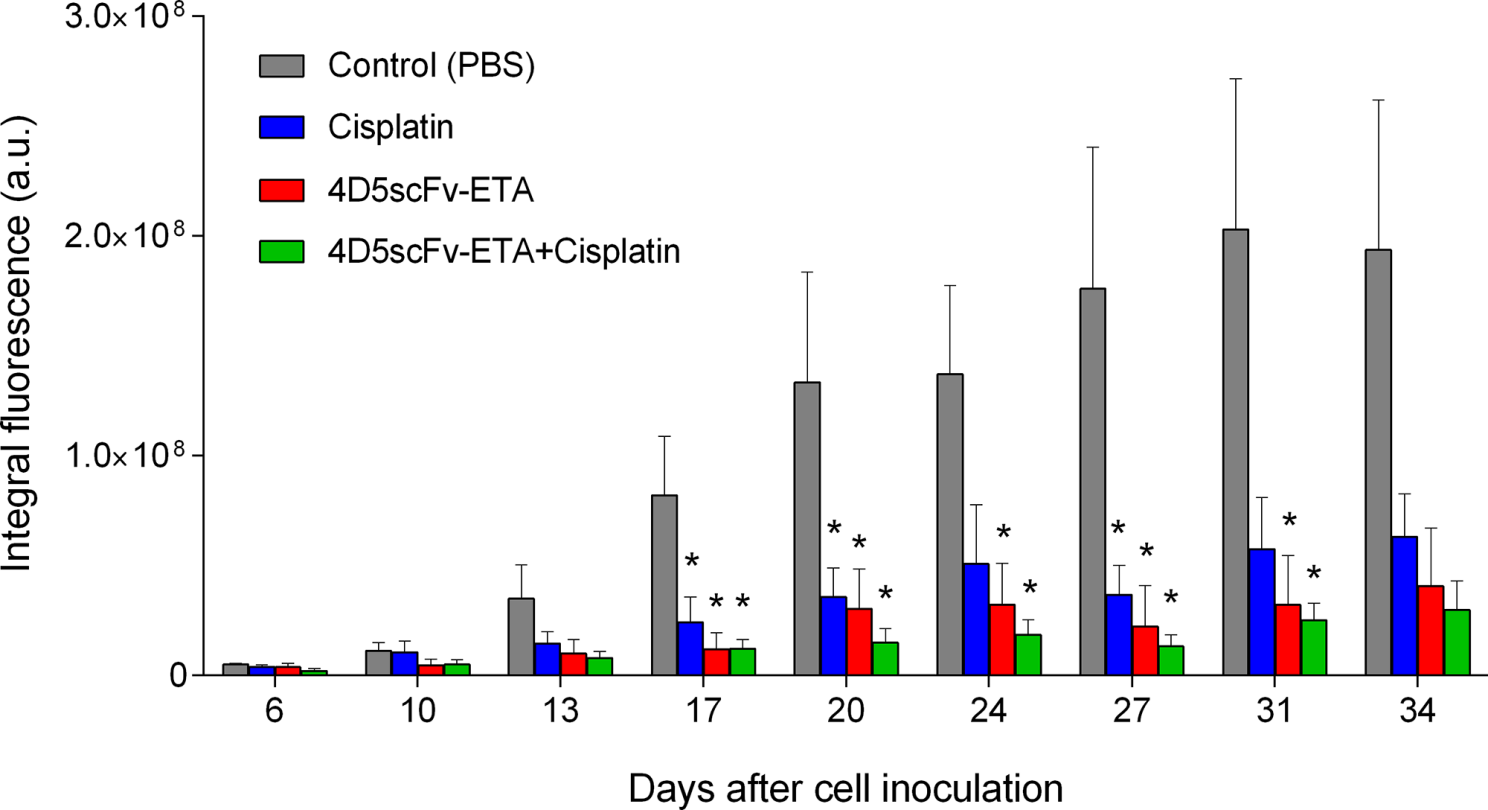
Tumor progression measured by *in vivo* fluorescent whole-body imaging in four groups of animals: groups treated with 4D5scFv-ETA, cisplatin, combination of 4D5scFv-ETA and cisplatin, and control group (PBS injection) The day of inoculation of SKOV-kat cells to animals was set as day 0. The data are represented as mean ±SEM. “*” indicates *a* value that reliably differs from the respective control value at *p* < 0.05 (Dunnett's test, *n* = 4–5).

Single injection of 4D5scFv-ETA resulted in a suppression of the tumor growth with the tumor growth inhibition coefficient of about 80% (Fig. 7, *red bars*). The combined treatment of tumors by 4D5scFv-ETA and cisplatin slightly enhanced the anti-tumor effect (Fig. 7, *green bars*). In this group the tumor growth inhibition coefficient reached 85%. Although a single administration of the immunotoxin-either alone or in combination with chemotherapy-had a pronounced anti-tumor effect, it was not sufficient for total elimination of tumor cells.

## DISCUSSION

Estimation of anti-tumor potential *in vivo* is an essential part of preclinical study of novel therapeutic agents, so development of appropriate animal tumor models has become a fast-growing field of experimental oncology. In recent decades opportunities of *in vivo* tumor visualization have been greatly expanded by means of fluorescent protein technology and optical fluorescence imaging methods that in combination provide long-term and highly sensitive whole-body monitoring of tumor growth. On the other hand, a tremendous evolution of targeted therapy of cancer demands a variety of tumor models expressing different molecular targets.

HER2 receptor is among the first successfully approved targets for solid tumors treatment. HER2-specific therapeutic antibodies (Trastuzumab, Pertuzumab) as well as antibody conjugate (Ado-Trastuzumab Emtansine) and tyrosine kinase inhibitor (Lapatinib) are now commercially available on the market. Huge number of potential HER2-targeted drugs are under clinical or preclinical studies.

In this study, we have created the first fluorescent xenograft model of the human tumor overexpressing HER2 receptor. The model can improve the efficiency of work on the creation of targeted drugs as well as its informativity due to possibility to non-invasively monitor the formation of regional and distant metastases.

The fluorescent protein Katushka used for generation of cell line SKOV-kat belongs to a family of far-red fluorescent proteins that are well-suited for the *in vivo* imaging as their emission spectrum falls within the “window of tissue transparency” [17]. A number of cancer cell lines expressing red or far-red fluorescent proteins providing the largest depths of fluorescence signal detection have been developed over the past decade, among which are colorectal, pancreas and epidermoid carcinomas, breast adenocarcinoma, Lewis lung carcinoma, etc. [4, 18–20]. The brightness of Katushka is seven- to tenfold higher than that of spectrally close protein HcRed or mPlum; besides, Katushka is characterized by fast maturation, as well as a high pH stability and photostability [21]. Comparison with other red and far-red fluorescent proteins demonstrated that Katushka features optimal combination of high brightness and far-red fluorescence and is therefore most favorable for visualization within living tissues [21, 22]. Feasibility of Katushka for *in vivo* visualization of the tumor response to treatment has been shown by Pardo and co-authors [18] as well as Rask and co-authors [19].

For the first proof of principle, the created model was used for *in vivo* evaluation of anti-tumor efficacy of chemotherapeutic agent cisplatin and novel HER2-targeted recombinant immunotoxin based on the scFv fragment of Trastuzumab (4D5scFv) as a targeting module and fragment of Pseudomonas exotoxin A (ETA) as a toxic module. Immunotoxins composed of functionally independent toxic and targeting modules are now believed to represent state-of-the-art therapeutic agents for the HER2-targeted therapy. We have previously designed several HER2-targeted immunotoxins with different mechanisms of action based on 4D5scFv: immunoRNAse [23] and immunophotosensitizers [24, 25]. It was shown that 4D5scFv is an extremely effective targeting module for HER2-targeted delivery.

*In vitro* experiments proved that 4D5scFv-ETA at picomolar concentrations eliminated HER2-overexpressing cells, including SKOV-3. Cytotoxicity of 4D5scFv-ETA significantly exceeded that of Trastuzumab [26] and was comparable with the cytotoxicity of other ETA-based anti-HER2 immunotoxins presented by other authors [27–29]. It should be noted that cell line SKOV-3 is known to be highly resistant to Trastuzumab and to many other cytotoxic drugs [30]. ETA used in this study negligibly decreased the viability of SKOV-3 cells. Combination of ETA with targeting anti-HER2 module greatly enhanced the cytotoxic efficacy of ETA with respect to these cells. The value of IC_50_ for 4D5scFv-ETA was almost two orders of magnitude lower than the value of IC_50_ for ETA. This effect was yet more pronounced for the Katushka-transfected SKOV-3-derived cells (SKOV-kat).

*In vivo* tumor growth monitoring on the SKOV-kat xenograft tumor model revealed a high therapeutic potential of tested anti-tumor agents with the tumor growth inhibition coefficient of about 80% for the immunotoxin and 85% for combined therapy with the immunotoxin and cisplatin. One possible approach to further enhance therapeutic efficacy of the immunotoxin is multiple-dose administration but it is limited by the immunogenicity of recombinant proteins based on bacterial toxins. These immunotoxins can induce the formation of neutralizing antibodies hindering the efficacy of immunotoxins [31]. Recently an approach for minimization of immunogenicity of ETA-based immunotoxins via elimination of B-cell and T-cell epitopes has been suggested [32, 33].

In conclusion, we have created the first fluorescent model of human HER2-overexpressing ovarian cancer. Due to far-red emission this model can be easily visualized and quantified by fluorescence imaging methods and should be highly advantageous for screening and preclinical study of different HER2-targeted drugs alone as well as in combination with commonly used chemotherapy.

## MATERIALS AND METHODS

### Generation of a fluorescent tumor cell line overexpressing HER2

The human ovarian adenocarcinoma SKOV-3 cell line was cultured in McCoy's 5A medium supplemented with 10% (v/v) fetal calf serum (HyClone) and 2 mM *L*-glutamine. Cells were grown in the humidified atmosphere with 5% CO_2_ at 37°C.

To obtain a fluorescent tumor cell line, SKOV-3 cells overexpressing HER2 were transfected with mammalian expression vector pTurboFP635-N (Evrogen) encoding the far-red fluorescent protein Katushka (commercial name TurboFP635) using Unifectin-56 transfection reagent (Unifectin Group) according to the manufacturer's protocols. Cells were selected in the medium containing 500 μg/ml of G418 (Sigma), and stably transfected cells were sterile sorted on a FACSVantage DiVa cell sorter (BD Biosciences, USA) equipped with a yellow 561 nm laser to excite TurboFP635. Cells were sorted, expanded in cell culture and sorted again to obtain the brightest fluorescent protein subset, with three sort/expansions cycles. The obtained cell line named SKOV-kat was maintained in the same way as parental cells SKOV-3.

### Xenograft tumor model

Six- to eight-week-old immunocompromised female nude mice (20–23 g) were purchased from the SPF (specified pathogen-free) licensed nursery of Shemyakin & Ovchinnikov Institute of Bioorganic Chemistry of the Russian Academy of Sciences. Animals were kept in well-ventilated polypropylene cages with a 12-h light-dark cycle, fed with sterilized standard laboratory food and supplied with water *ad libitum*. All experimental procedures were approved by the Animal Care and Use Committee of the Institute.

SKOV-kat cells grown in culture flasks were carefully detached using PBS (pH 7.4) with 5 mM EDTA, centrifuged, and resuspended in sterile PBS to a final concentration of 4 × 10^7^ cells/ml. Then the cell suspension was mixed with basement membrane-like matrix Matrigel (BD Biosciences, USA) at a ratio of 1:1 and the mixture was inoculated subcutaneously in the subscapular region (0.1 ml per mouse). The HER2 expression in the developing tumors was analyzed by HercepTest (Dako) according to the manufacturer's instructions. To confirm the TurboFP635 expression in the tumor xenografts, confocal microscopy of the excised non-fixed tissues was performed.

### Confocal microscopy

Stained cells and xenograft tumor tissues were imaged using an inverted laser scanning confocal fluorescence microscope Axiovert 200M LSM 510 META NLO (Carl Zeiss, Germany). The images were obtained with a 100× oil immersion objective with a numerical aperture of 1.4. Fluorescence of TurboFP635 protein was excited by the HeNe laser at 543 nm and collected in the range of 597–661 nm. To visualize Hoechst 33258 stained nuclei, Ti:Sapphire femtosecond tunable laser was used for two-photon excitation at 720 nm, and emission was collected in the range of 390–465 nm.

### Construction, expression and purification of immunotoxin 4D5scFv-ETA

The plasmid for expression of fusion protein 4D5scFv-ETA was constructed on the basis of the pSD-4D5-barnase plasmid [10]. Genetically engeneered manipulations, cell culturing, and cell lysis were performed according to standard protocols. The DNA fragment encoding ETA protein was amplified from plasmid pIG6-4D5MOCB-ETA [34] using primers 5′-actacGGCGCGCCGGAGTTCCCGAAACCGTCCAC and 5′-tgcgtAAGCTTCTACAGTTCGTCTTTATGGTG. The product of amplification was cloned into pSD-4D5-barnase plasmid instead of barnase gene using AscI and HindIII restriction endonucleases. The resulting construct pSD-4D5scFv-ETA was verified by sequencing.

For production of 4D5scFv-ETA containing His_6_-tag on *C-terminus*, the *Escherichia coli* expression strain BL21 was transformed with pSD-4D5scFv-ETA and grown in lysogeny broth (LB) at 28°C. Expression of 4D5scFv-ETA was induced by the addition of 0.5 mM IPTG at OD_550_ of 0.8. The bacteria were then incubated at 28°C for 12 h. The cells were harvested, centrifuged, and the pellet was resuspended in the lysis buffer (5 mM Tris-HCl, 40 mM K_2_HPO_4_, pH 8.3, with 0.5 M NaCl) and sonicated on ice. The lysate was then centrifuged at 22000 g for 30 min at 4°C. The pellet was used for purification of His_6_-tagged protein on Ni^2+^-NTA column (GE Healthcare) under the conditions recommended by the manufacturer. The protein was eluted with 250 mM imidazole. For final purification of 4D5scFv-ETA, elution fractions were diluted 20-fold, applied onto Q Sepharose FF 1-ml column (GE Healthcare) and eluted using linear gradient from 25 to 500 mM NaCl.

The SDS/PAGE analysis of the proteins was performed according to standard protocols using 12.5% polyacrylamide gels.

### Evaluation of the immunotoxin affinity

Measurements of the 4D5scFv-ETA dissociation constant were performed using a BIAcore 3000 analyzer (GE Healthcare). Recombinant p185^HER2-ECD^ (Sino Biological, Inc.) was coupled onto a CM5 chip at a density of 4500 RU by a standard amine coupling chemistry. Immunotoxin was used at four concentrations (1 μM, 330 nM, 110 nM, and 37 nM) in HBS-PE (0.1 M HEPES, pH 7.4, 0.15 M NaCl, 3 mM EDTA, 0.005% Tween-20). The sensograms were obtained at a flow rate of 5 μl/min at 25°C. The dissociation phase lasted 20 min.

### Cell treatment and cell viability assay

Cells SKOV-3, SKOV-kat, CHO (Chinese hamster ovary), and HeLa (human cervical carcinoma) were cultured in McCoy's 5A medium (for SKOV-3 and SKOV-kat) or RPMI-1640 (for HeLa and CHO) with 10% (v/v) fetal calf serum (HyClone) and 2 mM *L*-glutamine. Cells were grown in 5% CO_2_ at 37°C.

Cytotoxicity of 4D5scFv-ETA was estimated using MTT assay [35]. The cells were seeded in 96-well plates at a density of 4 × 10^3^ (SKOV-3, SKOV-kat, and HeLa) or 6 × 10^3^ (CHO) cells per well and were allowed to attach overnight. The medium was removed, and the cells were then incubated in the presence of 4D5scFv-ETA at different concentrations or with a control protein (free ETA or free 4D5scFv) in growth medium at 37°C for 72 h in 5% CO_2_. Then the cells were washed twice with PBS and incubated with serum-free medium containing 0.5 mg/ml MTT for 1 h. Formazan formed from the reduction of MTT was dissolved in DMSO, and the absorbance was measured at 540 nm with Synergy MX plate reader (BioTeck, USA). The amount of formazan produced is assumed to be proportional to the number of living cells. Cell viability was expressed as a ratio of the optical density of treated and untreated cells given in percents. Experiments were performed in triplicate and repeated at least two times. Data analysis and calculation of IC_50_ was performed using the GraphPad Prism 6 software.

### Estimation of anti-tumor efficacy in xenograft models: treatment schedule

All animals were divided into 3 groups (5 mice per group) according to the variant of the treatment.

4D5scFv-ETA was injected into a mouse intraperitoneally in a single dose of 50 pmol per animal in 0.1 ml PBS 24 h after the inoculation of tumor cells. In the control group mice were injected with 0.1 ml PBS.

Cisplatin (Cisplatin Ebewe, EBEWE Pharma GmbH Nfg KG, Austria) was administered at a single intraperitoneal dose of 200 μmol per mouse on day 3, 5 and 7 after the inoculation of tumor cells. Tumor fluorescence was measured twice a week by means of fluorescence imaging.

### *In vivo* whole-body fluorescence imaging

Whole-body *in vivo* fluorescence imaging was performed using a home-built back-reflectance imaging system (Institute of Applied Physics RAS, Russia) [36]. Fluorescence was excited by LED at a wavelength of 585 nm; for the emission collection, band-pass filter 628–672 nm was used. All fluorescence images were acquired with an exposure time of 2 s.

Fluorescence images were analyzed using ImageJ software (National Institute of Health, USA). The averaged signal was calculated in two regions of interest (ROI): tumor area and a region of the same area sited symmetrically relative to the animal spine (background). The resulting integral fluorescence signal was calculated as a difference between the fluorescence of the tumor and the background fluorescence. Data are presented as mean ±SEM. Statistical analysis was conducted using one-way ANOVA (one-way analysis of variance) and Dunnett's test (Primer of Biostatistics 4.03 software). The difference between compared values was considered statistically significant at *p* < 0.05.

To evaluate the efficacy of therapeutic agents under study, coefficient of tumor growth inhibition (TGI%) was calculated according to the following formula: TGI% = [(Fl_control_ – Fl_experiment_) × 100%]/Fl_control_, where Fl is an integral fluorescence intensity in the tumor area at a selected time point.

## SUPPLEMENTARY FIGURES


