# Supplementary material for: A novel far-red fluorescent xenograft model of ovarian carcinoma for preclinical evaluation of HER2-targeted immunotoxins
Source: Oncotarget. 2015 Sep 5;6(31):30919–28. doi: 10.18632/oncotarget.5130 (PMC4741577; doi:10.18632/oncotarget.5130)
Supplement: Supplementary file 1 [file oncotarget-06-30919-s001.pdf]

SUPPLEMENTARY FIGURES

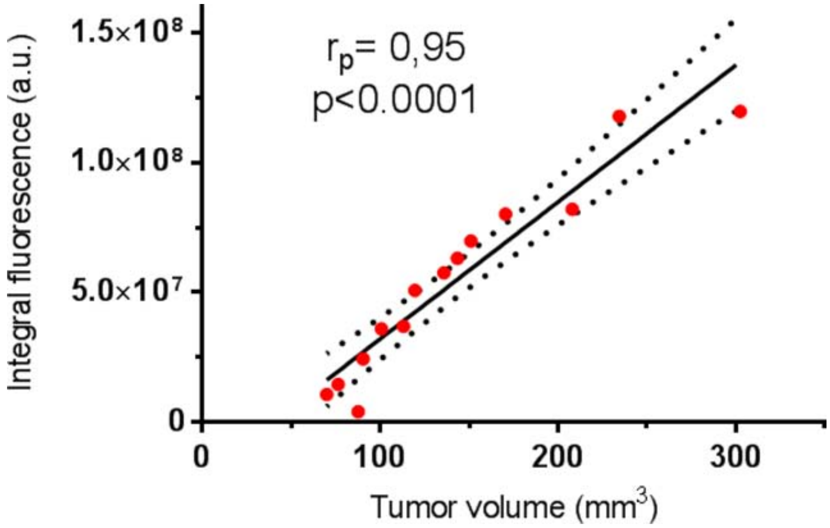

Supplementary Figure S1: The correlation of tumor volume calculated on vernier caliper measurements and integral fluorescence of tumor. Pirson correlation coefficient (r_p) and two-tailed *p*-value for 95%-confidence interval are presented. Dashed lines show 95%-confidence band for the best-fit line.

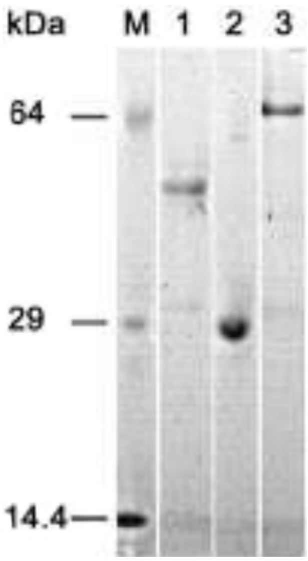

Supplementary Figure S2: 12.5% SDS-PAGE confirming the purification of ETA (lane 1, 41 kDa), 4D5scFv (lane 2, 29 kDa) and 4D5scFv-ETA (lane 3, 71 kDa). Coomassie Brilliant Blue R-25 stained gel. M, standard protein markers.

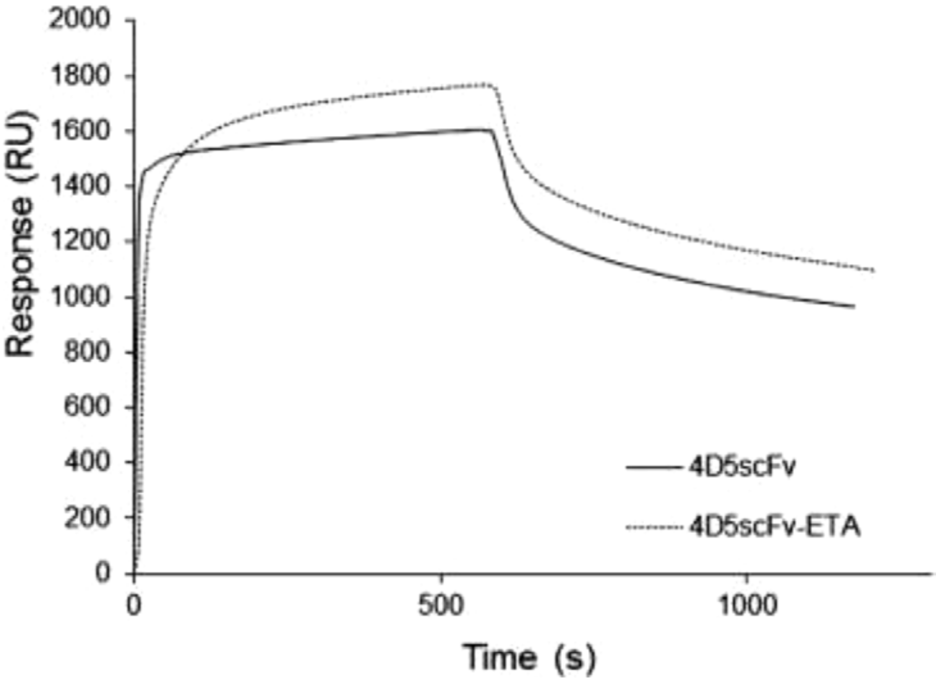

Supplementary Figure S3: Kinetics of 4D5scFv (solid line) and 4D5scFv-ETA (dashed line) measured by surface plasmon resonance (BIAcore). Proteins were used at 3 µM concentration.

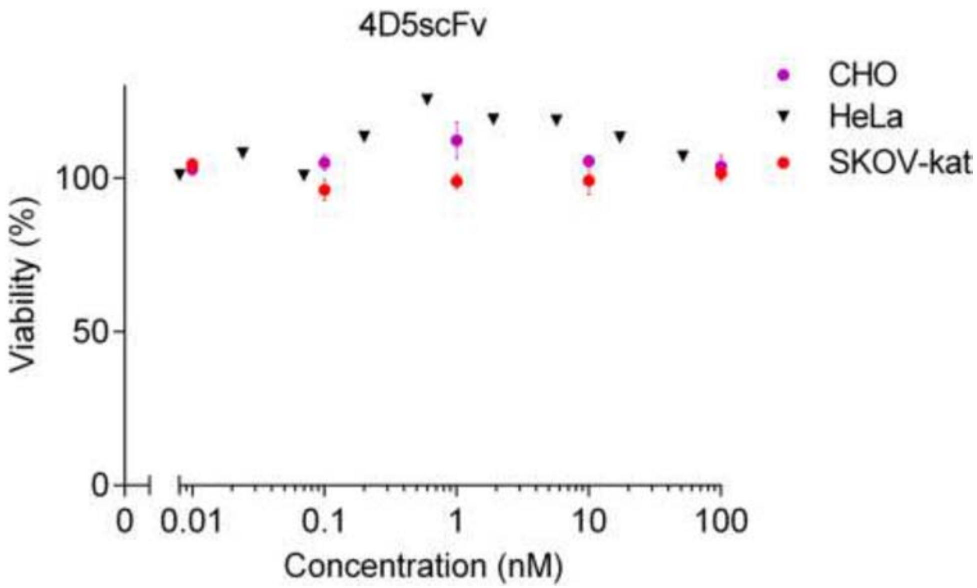

Supplementary Figure S4: *In vitro* analysis of the 4D5scFv-ETA cytotoxicity. Relative viability of HER2-positive cells SKOV-kat (red circles) and HeLa (black inverted triangles), and of HER2-negative cells CHO (magenta circles) after the treatment with free 4D5scFv at different concentrations. Error bars represent the standard error of the mean (SEM) of triplicate wells. The experiments were repeated at least 2 times.
